# Outcomes of pulsed-field ablation with low to zero fluoroscopy utilization for atrial fibrillation treatment

**DOI:** 10.1093/europace/euaf197

**Published:** 2025-09-01

**Authors:** Paul C Zei, David Newton, Devi Nair, Christopher F Liu, Mark D Metzl, Anshul M Patel, Luigi Di Biase, Jose Osorio, Moussa Mansour, Hugh Calkins, Oussama Wazni, Vivek Y Reddy, Andrea Natale, William H Sauer

**Affiliations:** Department of Medicine, Mass General Brigham and Harvard Medical School, 75 Francis Street, Boston, MA 02115, USA; Clinical Cardiac Electrophysiology, Memorial Health, Savannah, GA, USA; Cardiac Electrophysiology, St. Bernards Medical Centre & Arrhythmia Research Group, Jonesboro, AR, USA; Weill Cornell Medicine, New York Presbyterian Hospital, New York, NY, USA; Cardiac Electrophysiology, Endeavor Health, Glenview, IL, USA; Emory Arrhythmia Centre, Emory St. Joseph’s Hospital, Atlanta, GA, USA; Cardiac Arrhythmia Centre, Division of Cardiology at the Montefiore Medical Centre, Albert Einstein College of Medicine, New York, NY, USA; Cardiac Electrophysiology, HCA Florida Miami, Miami, FL, USA; Department of Medicine, Mass General Brigham and Harvard Medical School, Boston, MA, USA; Department of Medicine, Johns Hopkins Medical Institutions, Baltimore, MD, USA; Section Head, Cardiac Electrophysiology Service, Cleveland Clinic Foundation, Cleveland, OH, USA; Helmsley Electrophysiology Center, Mount Sinai Fuster Heart Hospital, New York, NY, USA; Clinical Cardiac Electrophysiology, Texas Cardiac Arrhythmia Research Foundation, Austin, TX, USA; Department of Medicine, Division of Cardiology, University of Tor Vergata, Rome, Italy; Department of Medicine, Mass General Brigham and Harvard Medical School, 75 Francis Street, Boston, MA 02115, USA

**Keywords:** Zero fluoroscopy, Low fluoroscopy, Pulsed-field ablation, PFA, Pulmonary vein isolation, Atrial fibrillation

Radiation use for medical procedures should be based on the guiding principle of ‘As Low As Reasonably Achievable (ALARA)’, which recommends minimizing or eliminating radiation use while maintaining efficacy and safety.^1^ Zero fluoroscopy (ZF) techniques have been successfully used for pulmonary vein isolation (PVI) for radiofrequency and cryothermal ablation of atrial fibrillation (AF).^2–4^ With increasing use of pulsed-field ablation (PFA),^5,6^ there is an interest in developing ZF workflows for PFA systems. The multicentre, single-arm AdmIRE study evaluated the safety and effectiveness of paroxysmal AF ablation with the variable-loop circular catheter (VLCC; VARIPULSE Catheter; Biosense Webster, Inc., part of Johnson & Johnson MedTech, Irvine, CA), a steerable, irrigated, multielectrode PFA delivery catheter integrated with an electroanatomic mapping (EAM) system (CARTO 3; Biosense Webster, Inc., part of Johnson & Johnson MedTech).^7^ This retrospective analysis evaluated the impact of fluoroscopy use on outcomes in AdmIRE.

The study methods, PFA system, parameters, and procedure have been described previously.^7–9^ Ethics committees at each centre approved the study; all included patients provided written informed consent. For this retrospective analysis, index ablations performed by 49 operators from 30 centres were segmented into three subgroups: ZF (0 min fluoroscopy), low fluoroscopy (LF; >0–≤5 min fluoroscopy), and conventional fluoroscopy (CF; >5 min fluoroscopy; *Figure 1*). For ZF or LF operators, intracardiac echocardiography (ICE) and EAM^10^ were used for guiding introduction of an EAM-visualizable, steerable sheath for mapping catheter and VLCC insertion.

**Figure 1:**
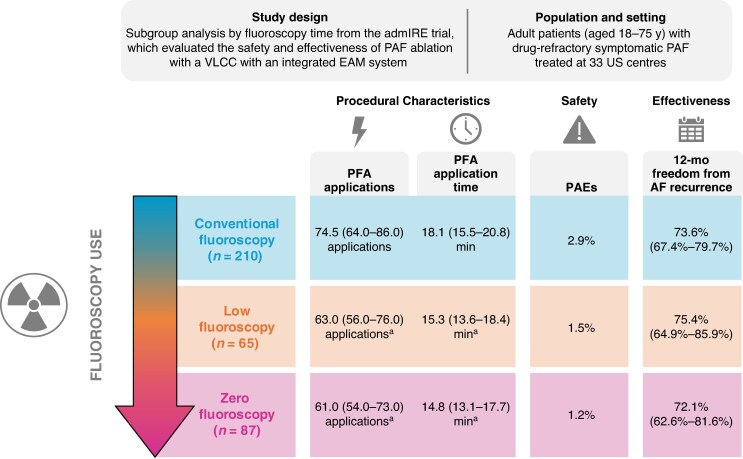
Study design and key procedural, effectiveness, and safety outcomes by fluoroscopy use from a retrospective analysis of the AdmIRE study. Data shown are median (IQR) for PFA applications and application time, percentages (95% CI) for 12-month freedom from recurrence, or percentages for PAEs. AF, atrial fibrillation; EAM, electroanatomic mapping; PAE, primary adverse event; PAF, paroxysmal AF; PFA, pulsed-field ablation; VLCC, variable-loop circular catheter. ^a^Significantly different from the conventional fluoroscopy group (*P* < 0.001). The figure is a courtesy of © Biosense Webster, Inc., part of Johnson & Johnson MedTech. All rights reserved.

Demographic, clinical, and procedural characteristics were evaluated by subgroup. Effectiveness was based on 12-month freedom from AF recurrence (Kaplan–Meier analysis). Safety was based on primary adverse event (PAE) occurrence.^7^ Differences across subgroups were tested using analysis of variance or the Kruskal–Wallis test for continuous variables and Fisher’s exact test for categorical variables. *Post hoc* tests were conducted using the Bonferroni correction or Dwass, Steel, Critchlow–Fligner method. Independent predictors of fluoroscopy use were assessed using binomial generalized linear regression models. As the decision to use ZF was at the physician’s discretion, ZF and LF cases were combined for multivariable analyses. Recognizing the potential clustering effect of patients treated by the same operator, a random intercept for operator was included in our model to account for unobserved operator-level variability. Significantly associated variables (*P* < 0.20) were considered for a multivariable model, and jointly significant variables (*P* < 0.05) were retained in a final multivariable model.

Of 362 patients at 30 US centres, 87 (24.0%; 8 sites), 65 (18.0%; 14 sites), and 210 (58.0%; 25 sites) patients were included in the ZF, LF, and CF subgroups, respectively. Operator experience with PFA varied, with 29.9%, 24.6%, and 61.4% of ZF, LF, and CF cases, respectively, performed by operators with high experience with PFA. Baseline demographics and medical history were comparable across subgroups; however, the proportion of patients with prior antiarrhythmic drug failure was lowest in the ZF (75.9%) followed by the LF (86.2%) and CF (92.4%; *P* < 0.001) subgroups.

Median [interquartile range (IQR)] fluoroscopy times were 2.7 (1.5–3.9) and 12.1 (8.0–20.0) minutes in the LF and CF subgroups, respectively. An EAM-visualizable, steerable sheath was used at a significantly higher rate in the ZF (82.8%) and LF (76.9%) subgroups vs. the CF subgroup (58.6%; *P* < 0.001). Median (IQR) total procedure time was significantly shorter in the ZF [79.0 (59.0–113.0) minutes] vs. the LF [96.0 (78.0–119.0) minutes] and CF [97.0 (72.0–127.0) minutes; *P* = 0.013] subgroups, despite a comparable lesion set in all subgroups (primarily PVI alone). Median valid PFA application time was shorter, and number of valid PFA applications was lower with ZF (14.8 min; 61.0 applications) and LF (15.3 min; 63.0 applications) vs. CF (18.1 min; 74.5 applications; both *P* < 0.001; *Figure 1*). Of note, with the VARIPULSE system, three applications are equivalent to one ablation. Nevertheless, rates of first-pass isolation trended higher with ZF (93.1%) and LF (95.4%) vs. CF (86.1%; *P* = 0.051).

In the ZF, LF, and CF subgroups, respectively, the study catheter was used for post-ablation mapping in 4.6%, 9.2%, and 21.9% of patients; a pentaspline mapping catheter was used in 95.4%, 90.8%, and 62.9% of patients; and ICE was applied in 100%, 84.6%, and 93.3% of patients. A lasso mapping catheter was only used in the CF (23.0%) subgroup.

In the final multivariable model, vascular disease was the only predictor of CF use and was associated with a 71.0% decrease in the odds of ZF or LF use (*P* = 0.065).

Eight subjects experienced nine PAEs, with no significant difference between subgroups (*Figure 1*). Vascular complications (*n* = 2) and pericardial effusion/tamponade (*n* = 3) were only reported in the CF subgroup. Other complications included transient ischaemic attack (*n* = 1; ZF subgroup), stroke/cardiovascular accident (*n* = 1 each; CF and LF subgroups), and pericarditis (*n* = 1; CF subgroup).

Twelve-month freedom from AF was 73.5% overall, with similar rates in the ZF (72.1%), LF (75.4%), and CF (73.6%) subgroups (*Figure 1*).

In this retrospective analysis of data from AdmIRE, 1-year freedom from recurrent AF was equivalent between the three subgroups, confirming that adoption of a ZF or LF approach with an EAM system–integrated PFA catheter maintains procedural effectiveness. Complication rates trended lower with ZF and LF vs. CF, although differences were not statistically significant. Number of PFA applications and PFA application time were lower with ZF and LF than with CF. In the ZF and LF subgroups, EAM and ICE allow for real-time visualization of catheter position, while integration of the VLCC into an EAM system permits improved spatial and temporal accuracy of catheter visualization, which may facilitate adoption of fluoroscopy-free techniques. This *post hoc* analysis of AdmIRE was subject to potential inherent biases, including the non-randomized nature of subgroup allocations; a tendency for ZF, LF, and CF to segregate with operators and centres; and differences in procedural workflow, operator approach, procedural heterogeneity, and mapping technology imbalance that could affect reconnection assessment. Twenty-five of 30 centres used CF workflows, indicating that centres that used CF workflows also had ZF or LF workflow options. Conventional fluoroscopy operators tended to be more experienced, suggesting higher effectiveness and lower complications with CF, although not seen in our data. To account for these confounders, our final multivariable model adjusted for random effect of operator-level variability. Non-significant differences were observed in key outcomes, including comparable complication rates across subgroups. As this analysis was not powered to detect significant differences between workflows, *P*-values should be considered hypothesis-generating rather than confirmatory. Further study is needed; however, these data suggest that LF and ZF strategies are safe and effective for AF ablation procedures, including those performed with pulsed-field energy.

## Data Availability

Johnson & Johnson MedTech has an agreement with the Yale Open Data Access (YODA) Project to serve as the independent review panel for the evaluation of requests for clinical study reports and patient-level data from investigators and physicians for scientific research that will advance medical knowledge and public health. Requests for access to the study data can be submitted through the YODA Project site at http://yoda.yale.edu.
